# Inflammation throughout pregnancy and fetal growth restriction in rural Nepal

**DOI:** 10.1017/S0950268819001493

**Published:** 2019-08-30

**Authors:** Michael W. Sauder, Sun Eun Lee, Kerry J. Schulze, Parul Christian, Lee S. F. Wu, Subarna K. Khatry, Steven C. LeClerq, Ramesh K. Adhikari, John D. Groopman, Keith P. West

**Affiliations:** 1 Department of Internal Medicine, Penn Medicine Lancaster General Health, Lancaster, PA, USA; 2 Department of International Health, Center for Human Nutrition, Johns Hopkins Bloomberg School of Public Health, Baltimore, MD, USA; 3 The Bill & Melinda Gates Foundation, Seattle, WA, USA; 4 Nepal Nutrition Intervention Project-Sarlahi, Kathmandu, Nepal, USA; 5 Department of Environmental Health & Engineering, Johns Hopkins Bloomberg School of Public Health, Baltimore, MD, USA

**Keywords:** Fetal growth restriction, laboratory tests, low birth weight, maternal inflammation, orosomucoid

## Abstract

Maternal systemic inflammation during pregnancy may restrict embryo−fetal growth, but the extent of this effect remains poorly established in undernourished populations. In a cohort of 653 maternal−newborn dyads participating in a multi-armed, micronutrient supplementation trial in southern Nepal, we investigated associations between maternal inflammation, assessed by serum *α*_1_-acid glycoprotein and C-reactive protein, in the first and third trimesters of pregnancy, and newborn weight, length and head and chest circumferences. Median (IQR) maternal concentrations in *α*_1_-acid glycoprotein and C-reactive protein in the first and third trimesters were 0.65 (0.53–0.76) and 0.40 (0.33–0.50) g/l, and 0.56 (0.25–1.54) and 1.07 (0.43–2.32) mg/l, respectively. *α*_1_-acid glycoprotein was inversely associated with birth size: weight, length, head circumference and chest circumference were lower by 116 g (*P* = 2.3 × 10^−6^), and 0.45 (*P* = 3.1 × 10^−5^), 0.18 (*P* = 0.0191) and 0.48 (*P* = 1.7 × 10^−7^) cm, respectively, per 50% increase in *α*_1_-acid glycoprotein averaged across both trimesters. Adjustment for maternal age, parity, gestational age, nutritional and socio-economic status and daily micronutrient supplementation failed to alter any association. Serum C-reactive protein concentration was largely unassociated with newborn size. In rural Nepal, birth size was inversely associated with low-grade, chronic inflammation during pregnancy as indicated by serum *α*_1_-acid glycoprotein.

## Introduction

Small birth size is a major risk factor for infant morbidity and mortality in low- and middle-income countries (LMICs). More than 20 million babies are born with low birth weight (LBW; <2500 g) each year [1]. The highest risk regions for neonatal mortality associated with LBW are in Africa and South Asia [2]. Major contributors to LBW are preterm birth (PTB; gestational age <37 weeks) and fetal growth restriction (FGR) leading to a newborn who is small for gestational age (SGA). Approximately 13.7 million preterm babies and 32.4 million SGA babies are born in LMICs each year, of whom 2.8 million are both preterm and SGA [3, 4].

Preterm birth, SGA and stillbirth in LMICs have been strongly associated with antenatal infection [5–7]. Yet, despite the extent of poor pregnancy outcomes in impoverished regions, most clinically detailed investigations of infection and inflammation during pregnancy have been conducted in economically developed countries [6]. In less resourced areas, investigating placental histology for evidence of infection is difficult, leaving a limited and less specific, but increasingly assessed, set of serum biomarkers of inflammation to evaluate influences of inflammation on pregnancy outcomes. Two serum biomarkers of inflammation are most commonly measured in population-based studies, *α*_1_-acid glycoprotein (AGP) and C-reactive protein (CRP). Both are acute-phase proteins whose concentrations reliably increase during infection and inflammation: CRP spikes in the early phase of infection while AGP rises more slowly and remains elevated during convalescence [8]. They are both part of the innate immune system whose overall activity is modified during pregnancy [9]. Serum CRP levels generally rise while serum AGP declines throughout normal pregnancy in well-nourished populations [10]. Insufficient research has examined gestational concentrations of these acute-phase proteins and birth outcomes in undernourished settings like rural South Asia. A study in Nepal found an inverse association between maternal serum AGP levels in the third trimester of pregnancy and infant birth weight [11]. However, no studies have examined associations of both these biomarkers throughout pregnancy with multiple anthropometric measures at birth. We hypothesised that since serum AGP and CRP are biomarkers of inflammation, their concentrations during pregnancy may reflect clinical or subclinical infection that may contribute to FGR and small infant size.

In 1999–2001, we conducted a five-arm, cluster-randomised, double-masked, controlled trial of daily antenatal micronutrient supplementation, starting in the first trimester, to improve birth outcomes and infant survival [12, 13]. As part of the nutrition and health assessment protocol, we measured maternal serum levels of AGP and CRP in the first and third trimesters of gestation followed by birth anthropometry. All five groups received a recommended dietary allowance of vitamin A, based on earlier evidence of benefit [14]. In the control group, serum AGP decreased and CRP increased as anticipated during pregnancy. A supplement containing folic acid, iron and zinc was associated with decreased AGP and CRP by the third trimester compared to the control, although this decrease did not occur with a multiple micronutrient supplement [13]. Birth weights increased among infants born to the women assigned to daily iron-folic acid and a multiple micronutrient supplement group [15]. The current study analyses serum AGP and CRP data from this trial, looking for associations between maternal inflammation in pregnancy and foetal growth, indicated by birth size and weight.

## Methods

### Study design and population

The Nepal Nutritional Intervention Project-Sarlahi-3 (NNIPS-3) study was the third trial conducted by the NNIPS Project in the rural southeast plains District of Sarlahi in Nepal from 1999 to 2001, described previously [12, 15]. Briefly, 14 185 married women of reproductive age living in 270 administrative wards were eligible for a 5-weekly pregnancy surveillance, of whom 4998 became pregnant, confirmed by a urine-test, and received one of five coded supplements based on their cluster (ward) of residence: (1) vitamin A alone (the control group), (2) vitamin A and folic acid, (3) vitamin A, folic acid and iron, (4) vitamin A, folic acid, iron and zinc or (5) multiple [15] micronutrients. In a baseline (first trimester) interview, data were collected based on women's age, parity, socio-economic status (SES), literacy, occupation, pregnancy history, substance abuse, recent morbidity and anthropometry (weight, height and left−mid−upper arm circumference). Nutritional and health risk factor assessments were repeated in the third trimester [12].

In a subset of 81 wards balanced across the study area, consenting mothers participated in a substudy involving biochemical assessment at each trimester visit [12]. As presented in the figure, a total of 1361 substudy women were eligible for enrollment, of whom 1153 had data from a single live birth or still birth outcome, and 730 contributed blood samples from both the first and third trimester. Forty-three mothers gave birth to infants whose anthropometry was measured more than 72 h after birth, and were thus excluded. Another 34 mothers refused, migrated or had their infant die prior to birth size assessment, and were also excluded. Thus, 653 mother−infant dyads were eligible for the current analysis.

### Newborn anthropometric assessment

Anthropometric measurements were obtained as soon as possible after birth, and within 72 h; the median interval was 10.8 h after birth. Birth weight was measured to the nearest 2 g using a digital scale (Seca 727, Hamburg, Germany). Recumbent length was determined to the nearest 0.1 cm on a length board (Shorr Infant Measuring Board, Shorr Productions, Rhode Island, USA). Head and chest circumference were measured to the nearest 0.1 cm using an adult middle upper arm circumference insertion tape (Ross Laboratories, Columbus, Ohio, USA). For all measurements except weight, the median of three values was used in the statistical analyses.

### Assessments of serum inflammatory markers

The mean (s.d.) gestational ages at the first- and third trimester blood draws were 9.9 (4.1) and 32.3 (3.9) weeks, respectively. Venous blood was collected into 7-ml trace metal-free vacuum test tubes (Vacutainer; Becton Dickinson Company, Franklin Lakes, NJ) at home by trained phlebotomists. The blood was kept on a frozen cold pack, and brought to the project laboratory for centrifugation at 750 × ***g*** for 20 min to separate the serum. Aliquots of serum were stored in liquid nitrogen tanks in trace element-free cryotubes (Nalgene Company, Sybron International, New York, NY) and shipped to the Johns Hopkins Bloomberg School of Public Health in Baltimore, MD, where they were stored at −80 °C until analysis. AGP, expressed in g/l, was measured with a radial immunodiffusion assay with commercially available kits (Kent Laboratories, Bellingham, WA), and CRP, in mg/l, was measured by ELISA with a commercial kit from ADI (San Antonio, TX). Retinol, zinc and transferrin receptor were also measured, as previously described [16].

### Statistical analyses

Distributions of birth measurements and serum inflammation markers were evaluated for normality. Maternal baseline characteristics were compared between women included and women excluded in the study using chi-square tests. Gestational age and anthropometric measures of size at birth were compared between male and female newborns using two-sample *t*-tests. Relationships between maternal serum inflammation markers in the first, third and averaged trimester values, and birth size (birth weight, length, head circumference and chest circumference) were examined using generalised estimating equations. We specified the identity link function for Gaussian distribution of dependent variables and used an exchangeable correlation structure to account for the cluster effect associated with sector *vs.* individual randomisation in the antenatal supplementation. Because the AGP and CRP concentrations were skewed and a 1 g/l change in AGP is physiologically unlikely, we modelled the relationship between inflammatory markers and birth size as log-linear. We used log_2_ transformed AGP and CRP concentrations as independent variables, allowing birth size outcomes to be interpreted as differences per 50% increase in concentrations of AGP and CRP. For example, the mean difference in birth weight is interpreted as ‘x’ g per 50% increase in serum AGP concentration, whether, for example, the difference in AGP is 0.60−0.90 g/l or 1.0−1.5 g/l. We included maternal age, parity, mid-upper arm circumference (MUAC), serum retinol, zinc and transferrin receptor at baseline, literacy, household SES (radio ownership and caste), season at time of each blood draw, gestational age at birth to distinguish birth size attributable to fetal growth restriction *vs.* due to duration of gestation, and antenatal micronutrient supplementation arm (*n* = 5) in the fully adjusted models to control for possible confounding factors. Data were analysed using SAS and the R Environment for Statistical Computing (version 3.1.2. R foundation for Statistical Computing, Vienna, Austria). The dataset of maternal and household characteristics and infant birth outcomes reported in this study is available in a Supplementary Table.

## Results

Table 1 summarises and compares the characteristics of women included and excluded from the analysis. Both groups were comparable with respect to parity and the proportion who were thin (represented by a MUAC < 21.5 cm). However, included women were younger, and also of higher socio-economic standing, reflected by caste, ownership of a radio and reported literacy. Among those studied, approximately half were between 20–29 years old; parity was widely distributed with 27% nulliparous and 35% having previously had 3 or more live born children; and 31% of women were literate and 42% were thin, comparable to previous assessments in the southern plains of Nepal [14].

**Table 1.** Characteristics of Nepalese women in early pregnancy

	Women included (*N* = 653)		Women excluded (*N* = 708)^a^	
	*N*	%	*N*	%
Maternal age*				
<20	184	28.4	201	28.7
20–29	363	56.0	347	49.5
⩾30	101	15.6	153	21.8
Maternal parity				
0	176	27.0	181	26.2
1–2	248	38.0	246	35.7
⩾3	229	35.1	263	38.1
MUAC at baseline				
<21.5 cm	277	42.4	263	40.2
SES variables				
High-caste*	154	23.6	81	11.8
Radio in household*	240	36.8	215	31.2
Mother literate*	200	30.6	138	20.0

SES, socio-economic status; MUAC, middle upper arm circumference.

Five age values missing in included women, and 7 age values missing in excluded women.

High caste: Brahmin or Chhertri

a Women were excluded if they had miscarriage, multiple births, missing birth outcome data, no serum AGP and CRP data in either first or third trimester, or infant anthropometry measured >72 h of birth.

*P < 0.05, using *χ*^2^ test.

Inflammation was minimal in the first trimester of pregnancy, reflected by median (IQR) serum AGP and CRP concentrations of 0.65 (0.53, 0.76) g/l and 0.56 (0.25, 1.54) mg/l, respectively (Table 2). Only 5.2% and 6.4%, respectively, had values above conventional clinical cut-offs 1.0 g/l and 5.0 mg/l (not shown) [17]. The median values of serum AGP and CRP concentrations in the third trimester were 0.40 g/l and 1.07 mg/l, respectively, representing a 39% mean decrease in AGP and a 191% mean increase in CRP concentrations from the first trimester. Serum retinol increased during pregnancy (all women received a daily supplement of vitamin A). Serum zinc decreased while transferrin receptor increased.

**Table 2.** Maternal serum biomarker concentrations in Nepalese women during pregnancy (*N* = 653)

	First TM	Third TM
*α*1-acid glycoprotein (g/l)	0.65 (0.53, 0.76)	0.40 (0.33, 0.50)
C-reactive protein (mg/l)	0.56 (0.25, 1.54)	1.07 (0.43, 2.32)
Retinol (μmol/l)	1.15 (0.92, 1.39)	1.32 (1.09, 1.61)
Zinc (μg/l)	530 (447, 613)	434 (363, 509)
Transferrin receptor (μg/ml)	4.68 (3.35, 6.66)	5.08 (3.87, 7.42)

One value missing for transferrin receptor in the first trimester (TM).

Values are median (IQR).

Mean (s.d.) gestational age at birth was 39.1 (2.9) weeks (Table 3), with 14% of infants born preterm (<37 weeks, not shown). Birth weight was 2.67 (0.44) kg, with 36% of infants born below 2.5 kg (not shown). Females had a lower mean birth weight than males (*P* = 0.019).

**Table 3.** Gestational age and anthropometric measures of infant size at birth

	Male (*N* = 335)	Female (*N* = 318)	Overall (*N* = 653)
	Mean (s.d.)	Mean (s.d.)	Mean (s.d.)
Gestational age (weeks)	38.8 (3.0)	39.3 (2.8)	39.1 (2.9)
Weight (kg)	2.71 (0.45)	2.63 (0.42)*	2.67 (0.44)
Length (cm)	48.0 (2.1)	47.4 (2.0)	47.7 (2.0)
Head circumference (cm)	33.0 (1.3)	32.5 (1.3)	32.7 (1.3)
Chest circumference (cm)	31.0 (1.8)	30.7 (1.8)	30.9 (1.8)

s.d. standard deviation.

*P = 0.019 using a two-sample *t*-test.

The association between maternal AGP and newborn size metrics are summarised in Table 4. All regression coefficients yielded results of comparable magnitude and statistical significance, both unadjusted and adjusted for multiple maternal nutrition, health, demographic and socio-economic factors. In the first trimester an elevated maternal serum AGP concentration was inversely associated with all the infant size measures except head circumference, revealing an unadjusted 59 g lower birth weight (95% CI −105 to −12), a 0.21 cm (95% CI −0.41 to 0) shorter length and 0.23 cm (95% CI −0.40 to −0.06) smaller chest circumference per 50% increase in serum AGP concentration. Generally, larger decrements in newborn size were observed with rising AGP in the third trimester, when a 50% increase in serum AGP was associated with an unadjusted 85 g (95% CI −120 to −51) decrease in birth weight, a 0.35 cm (95% CI −0.52 to −0.17) decrease in length, a 0.17 cm (95% CI −0.29 to −0.05) decrease in head circumference and a 0.39 cm (95% CI −0.53 to −0.25) decrease in chest circumference.

**Table 4.** Associations between maternal serum AGP during pregnancy and infant birth size (*n* = 653)

	Unadjusted		Adjusted	
	Estimate (95% CI)^a^	*P*-value	Estimate (95% CI)^a^^,^^b^	*P*-value
First trimester^c^				
Weight, g	−59 (−105 to −12)	0.0131	−77 (−127 to −26)	0.0029
Length, cm	−0.21 (−0.41 to 0.00)	0.0447	−0.31 (−0.54 to −0.08)	0.0091
Head cir, cm	−0.05 (−0.19 to 0.08)	0.4393	−0.11 (−0.26 to 0.05)	0.1720
Chest cir, cm	−0.23 (−0.40 to −0.06)	0.0079	−0.25 (−0.45 to −0.05)	0.0125
Third trimester				
Weight, g	−85 (−120 to −51)	1.32 × 10^−6^	−78 (−113 to −43)	1.10 × 10^−5^
Length, cm	−0.35 (−0.52 to −0.17)	1.00 × 10^−4^	−0.32 (−0.49 to −0.15)	3.00 × 10^−4^
Head cir, cm	−0.17 (−0.29 to −0.05)	0.0059	−0.16 (−0.28 to −0.04)	0.0094
Chest cir, cm	−0.39 (−0.53 to −0.25)	2.50 × 10^−8^	−0.37 (−0.50 to −0.23)	1.70 × 10^−7^
Average				
Weight, g	−116 (−164 to −68)	2.30 × 10^−6^	−125 (−176 to −74)	1.70 × 10^−6^
Length, cm	−0.45 (−0.67 to −0.24)	3.10 × 10^−5^	−0.50 (−0.74 to −0.27)	1.90 × 10^−5^
Head cir, cm	−0.18 (−0.33 to −0.03)	0.0191	−0.21 (−0.38 to −0.05)	0.0117
Chest cir, cm	−0.48 (−0.66 to −0.30)	1.70 × 10^−7^	−0.48 (−0.69 to −0.28)	4.30 × 10^−6^

a Change in infant birth size per 50% increase in maternal serum AGP (g/l) concentration.

b Adjusted for maternal age, parity, baseline MUAC, baseline serum retinol, zinc, and transferrin receptor concentrations, literacy, radio ownership, high caste, season at time of each blood draw (first and third trimesters), gestational age at birth, and antenatal micronutrient supplementation. Data were missing for maternal age (*n* = 5), baseline MUAC (*n* = 5), and baseline serum transferrin receptor (*n* = 1).

c Log2 transformed AGP value was not available (*n* = 1).

The largest decrements in birth size were observed with an elevated mean serum AGP concentration across both trimesters, whereby a 50% increase was associated with an unadjusted 116 g decrease in birth weight (95% CI −164, −68), a 0.45 cm (95% CI −0.67, −0.24) decrease in length, a 0.18 cm (95% CI −0.33, −0.03) decrease in head circumference and a 0.48 cm (95% CI −0.66, −0.30) decrease in chest circumference.

First-trimester serum CRP was not associated with any newborn size dimension (Table 5). During the third trimester a 50% increase in serum CRP concentration was associated with a small (~0.04 cm) decrement in chest circumference in both the adjusted and unadjusted models. A 50% higher mean CRP concentration across the two trimesters was also associated with a small reduction in birth weight (−13 g). None of the changes associated with average CRP concentration were significant in the adjusted model.

**Table 5.** Associations between maternal serum CRP during pregnancy and infant birth size (*n* = 653)

	Unadjusted		Adjusted	
	Estimate (95% CI)^a^	*P*-value	Estimate (95% CI)^a^^,^^b^	*P*-value
First trimester				
Weight, g	−3 (−14 to 8)	0.6091	−1 (−12 to 10)	0.9016
Length, cm	0.02 (−0.03 to 0.07)	0.4445	0.04 (−0.02 to 0.09)	0.1698
Head cir, cm	0.01 (−0.02 to 0.05)	0.4646	0.02 (−0.02 to 0.05)	0.2762
Chest cir, cm	−0.02 (−0.06 to 0.03)	0.4939	−0.01 (−0.05 to 0.04)	0.7203
Third trimester				
Weight, g	−6 (−16 to 3)	0.1872	−8 (−17 to 2)	0.1079
Length, cm	−0.03 (−0.08 to 0.02)	0.2426	−0.03 (−0.08 to 0.02)	0.1782
Head cir, cm	−0.01 (−0.05 to 0.02)	0.3810	−0.02 (−0.05 to 0.01)	0.2610
Chest cir, cm	−0.04 (−0.08 to 0)	0.0466	−0.05 (−0.09 to −0.01)	0.0216
Average				
Weight, g	−13 (−25 to −1)	0.0360	−10 (−23 to 2)	0.0898
Length, cm	−0.04 (−0.1 to 0.03)	0.2636	−0.02 (−0.08 to 0.04)	0.5185
Head cir, cm	−0.02 (−0.05 to 0.02)	0.4358	−0.01 (−0.05 to 0.03)	0.6783
Chest cir, cm	−0.06 (−0.12 to −0.01)	0.0214	−0.05 (−0.1 to 0)	0.0561

a Change in infant birth size per 50% increase in maternal serum CRP (mg/l) concentration.

b Adjusted for maternal age, parity, baseline MUAC, baseline serum retinol, zinc, and transferrin receptor concentrations, literacy, radio ownership, high caste, season at time of each blood draw (first and third trimesters), gestational age at birth and antenatal micronutrient supplementation. Data were missing for maternal age (*n* = 5), baseline MUAC (*n* = 5), and baseline serum transferrin receptor (*n* = 1).

## Discussion

Foetal growth restriction is a profound public health problem, particularly in South Asia where it affects half of all live born infants and contributes to excessive infant and childhood morbidity, mortality and developmental delay [2, 18]. Among factors that contribute to FGR are maternal protein and energy malnutrition, micronutrient deficiencies, placental insufficiency and inflammation that could be caused by a variety of factors, including bacterial and viral infections and other environmental exposures [19]. However, risk relationships between maternal inflammation and FGR in rural communities of South Asia remain poorly quantified due to a paucity of population studies of sufficient size that relate inflammation throughout pregnancy to birth size. This study revealed a strong inverse association between antenatal serum concentration of AGP and newborn size. Unadjusted effect sizes were indiscernible from estimates adjusted for maternal age, parity, nutrition (first-trimester arm circumference, serum concentrations retinol, zinc and transferrin receptor and micronutrient supplementation allocation in the trial), caste/SES, season of each blood draw and gestational age at birth. The association was present in the first trimester, stronger in the third trimester and strongest when AGP concentrations were averaged across both trimesters, whereby a 50% increase in AGP was associated with a 125 g lower birth weight, 0.50 cm shorter length and 0.21 and 0.48 cm deficits, respectively, in head and chest circumferences. These findings imply that prolonged inflammation as represented by elevated AGP at any time of pregnancy, but especially that which persists throughout pregnancy, may restrict foetal growth, acting via pathways independent of maternal biological age and nutritional status, length of gestation, season and household SES. On the other hand, acute inflammation as indicated by elevated CRP at either trimester, or averaged across trimesters, had only little association with newborn size.

The levels of AGP in our study are similar to those previously noted in studies of pregnant women [20–22], including another in Nepal [11]. In contrast to CRP, AGP normally decreases over pregnancy [10, 20], as our study also observed. However, the precise functions of AGP, especially in relation to pregnancy, remain obscure, despite it being a major component of normal plasma (1–3% of total plasma protein) whose concentration can increase up to 10-fold during inflammation [23]. Best known as a lipocalin transport protein, AGP is also known to exert immunomodulatory and barrier functions. It down-regulates neutrophil migration and activation, in vitro [24, 25] and in vivo [26]. In its barrier function it binds to the phospholipid bilayer of plasma membranes of many different types of cells [23]. During infection, AGP may inhibit bacteria and neutrophils from attaching to cells [27, 28], and it also helps maintain capillary permeability during inflammation [24, 29]. Some functions of AGP seem pro-inflammatory (inducing some pro-inflammatory cytokines locally) but overall its anti-inflammatory effects predominate [24, 28]. A sustained, higher AGP concentration may thus be reflecting a continuous process of attempts to resolve repeated or sustained infectious processes during pregnancy.

Our results, which reveal a smaller birth size correlated with elevated AGP during pregnancy, strengthen the hypothesis that this protein is involved in regulating complex immune interactions between mother and foetus during pregnancy. In rats, AGP has been shown to be concentrated in the decidua capsularis of the placenta in early pregnancy, and may be involved in controlling immune interactions during implantation [30, 31]. In humans, AGP concentration in maternal blood, amniotic fluid and foetal blood, has been examined at various times during gestation [32]. There was no correlation between mother's and fetus' AGP levels, but there was a correlation between maternal and amniotic fluid AGP levels, possibly reflecting an elevation of AGP levels in response to placental infection, which has been shown to contribute to FGR [6]. AGP has not been studied as a marker for FGR, but the fact that in our study maternal serum AGP is so robustly associated with infant birth size suggests it should be investigated further as a marker of mothers at risk of bearing a low birth weight infant.

CRP is more commonly studied, especially in relation to cardiovascular disease in the developed world, but also in pregnancy. During pregnancy serum CRP becomes elevated as early as the 4th week [33], and can be deposited in the placenta during disease states [34]. Serum CRP may predict preeclampsia [35], and may be a risk marker for preterm birth, perhaps by reflecting intrauterine infection [36]. But as a predictor of FGR, only inconsistent associations have been found [37, 38]. A recent prospective study documented that high maternal CRP predicted lower birth weight, but did not adjust for gestational age at birth [39]. In our study, CRP levels were associated with only one birth size outcome, a smaller chest circumference.

Our study is distinct in the literature because two markers of inflammation were measured in both first and third trimesters and assessed for their potential influence on FGR, a burden affecting more than 40% of live born infants in South Asia [4]. The greater strength of association of an elevated AGP averaged across trimesters with smaller birth size, in contrast to CRP, reinforces a generally ascribed role of AGP as a marker of more prolonged inflammation [8], which may be more homeostatically disturbing than acute inflammation. Notably, at 6−8 years of age, among children born to these study mothers, we observed more acute and chronic phase proteins associated with plasma AGP (*n* = 99) than CRP (*n* = 81) [40], possibly reflecting greater metabolic alteration.

**Fig. 1.**
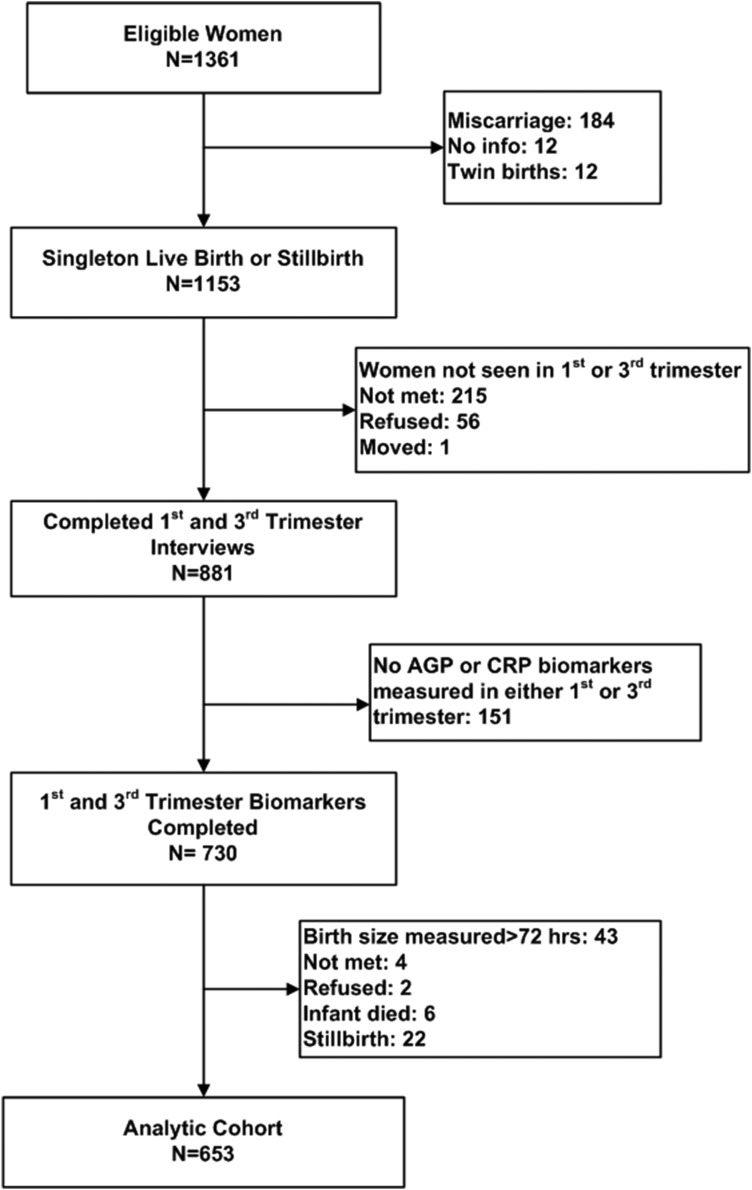
Consort diagram of study participants. AGP, alpha-1-acid glycoprotein; CRP, C-reactive protein.

A limitation of the study was that we had complete first- and third trimester biomarker and infant size data from only 653 (48%) of the original 1361 women. Missing values were 95% due to miscarriage, women transferring to their parental home for third trimester care, failure to measure AGP or CRP, newborn anthropometry obtained after 36 h, perinatal death or refusal (Fig. 1). Women with missing data tended to be older, and of both lower SES and caste (Table 1), factors that, coupled with perinatal deaths experienced by some missed women, would lead to an expectation of more morbidity and stronger associations between maternal AGP and smaller newborn size among excluded women.

In summary, in this rural South Asia population, we revealed a strong inverse association between higher maternal serum AGP and growth restricted, small newborn size. Little association was observed with CRP. In similar populations, assessing maternal AGP during pregnancy may be an important, predictive biomarker of impaired intrauterine growth.

## Supplementary material

For supplementary material accompanying this paper visit https://doi.org/10.1017/S0950268819001493. click here to view supplementary material
